# Progesterone Luteal Support in Natural Cycles for Unexplained Infertility: A Randomised Controlled Trial (The PiNC Trial)

**DOI:** 10.1111/1471-0528.18171

**Published:** 2025-04-21

**Authors:** Claudia Raperport, Elpiniki Chronopoulou, Aviva Petrie, Roy Homburg, Elizabeth Timlick, Sheetal Barhate, Kristina Sackett, Priya Bhide

**Affiliations:** ^1^ Women's Health Research Unit Wolfson Institute of Population Health, Queen Mary University of London London UK; ^2^ Whittington Health NHS Trust London UK; ^3^ Centre for Reproductive and Genetic Health London UK; ^4^ University College London London UK; ^5^ University College London Hospital NHS Foundation Trust London UK; ^6^ Homerton Healthcare NHS Foundation Trust London UK; ^7^ Barts Health NHS Trust London UK; ^8^ Fertility Plus London UK

**Keywords:** luteal phase defect, progesterone, unexplained infertility

## Abstract

**Objective:**

To compare the effect of luteal‐phase progesterone supplementation in natural cycles to expectant management on live birth rates in women with unexplained infertility (UI).

**Design:**

An open‐label, parallel‐arm, single‐centre randomised controlled trial.

**Setting:**

One tertiary NHS‐funded fertility unit.

**Population:**

Couples with UI for at least 1 year.

**Method:**

A comparison of luteal phase micronised vaginal progesterone treatment (400 mg bd) with timed intercourse and timed intercourse alone for 3 cycles.

**Main Outcome Measures:**

Primary outcome: Livebirth rate. Secondary Outcomes: Biochemical pregnancy, clinical pregnancy, mid‐luteal serum progesterone and pregnancy loss.

**Results:**

One hundred and forty‐three couples were randomised. Livebirth rates were 11/72 (15.3%) in the treatment group versus 5/71 (7.0%) in the control group (RR 2.17, 95% CI 0.79–5.93). Biochemical pregnancy rates were 15/72 (20.8%) versus 10/71 (14.1%), (RR 1.48, 95% CI 0.72–3.07) and clinical pregnancy rates were 14/72 (19.4%) versus 9/71 (12.7%), (RR 1.53, 95% CI 0.71–3.31) in the treatment and control groups respectively. Pregnancy losses: 4/15 treatment group versus 5/10 control group (RR 0.53, 95% CI 0.19–1.51). One biochemical pregnancy loss in each group and 2/15 miscarriages in the treatment group versus 3/10 in the control group. Total miscarriage rates including biochemical losses were 3/15 (20%) versus 4/10 (40%) (RR 0.5, 95% CI 0.14–1.77).

**Conclusions:**

The results did not reach statistical significance. However, the difference in livebirth rates warrants further investigation as this simple, noninvasive, inexpensive treatment would be a very attractive option for affected couples. A larger trial using the effect size from this study to guide sample size is planned.

**Trial Registration:**

The PiNC trial was registered with the EU Clinical Trials Register on 29/11/2019 www.clinicaltrialsregister.eu/ctr‐search/search?query=homerton. The first participant was recruited on 25/2/2020.

## 1 Introduction

Unexplained infertility (UI) accounts for 25%–50% of all couples with infertility [1]. It is a term applied to heterosexual couples who have been trying to conceive through regular unprotected sexual intercourse for at least 12 months despite a normal fertility assessment, usually including but not limited to semen analysis (SA), tubal patency and ovulation assessment [2]. Subsequently, the treatment options for UI are empirical and include expectant management, in vitro fertilisation (IVF) or intrauterine insemination (IUI). The National Institute for Health and Care Excellence (NICE) recommends that women with UI who have not conceived after 2 years of having regular unprotected sex should be offered IVF treatment [3]. In a more recent guideline, the European Society for Human Reproduction and Embryology (ESHRE) recommends IUI before offering IVF [1]. IUI and IVF are not directed at treating causality, are expensive, invasive, with limited access globally and report a suboptimal and relatively static success rate for the last decade. Despite this, no therapeutic options aimed at treating causality have been developed.

The underlying aetiology of UI is likely to be multifactorial, and although not ascertained by current standard investigations, may include embryonic and/or endometrial factors. Progesterone is the hormone associated with secretory transformation of the endometrium and key to endometrial receptivity. It is produced by the corpus luteum from ovulation until the pregnancy can produce enough to self‐support, from roughly 10–12 weeks of gestation.

A decrease in the production of progesterone or a reduced response to progesterone could impact the likelihood of successful implantation of an embryo during the luteal phase of the menstrual cycle. A recent systematic review reported on the role of endogenous progesterone in couples with UI [4]. It reported various outcomes related to the actions of endogenous progesterone in natural menstrual cycles. Although no differences were reported in mid‐luteal phase serum progesterone concentrations, which is the standard test included as a part of routine investigations in clinical practice, it reported a reduction in multiple downstream mediators and end‐organ measures of progesterone activity and endometrial receptivity, with a consistent direction of effect. The review highlights the subtle defects that are missed by routine clinical testing and may contribute to the cause of UI. Some indirect evidence from a recent systematic review that assessed the impact of luteal phase progesterone supplementation in natural cycle frozen embryo replacement reported an increased live birth rate [5]. This again highlights the possible subtle defects in the luteal phase of a broadly similar infertile population and improved outcomes with exogenous progesterone supplementation. This work led us to consider the hypothesis that exogenous progesterone alone could benefit couples trying to conceive in otherwise natural cycles.

Although this is not a causal factor in all couples with UI, supplementation of the luteal phase of natural, otherwise unmedicated menstrual cycles with exogenous progesterone may provide causality‐directed treatment for a subset of couples with UI. We hence hypothesise that this intervention may improve live birth rates in this group as compared to expectant management.

## 2 Objective

The aim of this trial was to investigate whether luteal phase support with exogenous progesterone could improve the live birth rates for couples with UI in otherwise natural cycles.

The objective was to conduct an open‐label, parallel‐arm randomised controlled trial (RCT) to evaluate the clinical effectiveness of exogenous luteal phase progesterone supplementation compared to no progesterone supplementation in natural cycles to improve live birth rates in couples with UI.

## 3 Methods

### 3.1 Study Design

Progesterone in Natural Cycles (PiNC) is a prospective, single‐centre, open‐label, parallel‐arm RCT of an investigational medicinal product (CTiMP). The trial was approved by the National Research Ethics Committee (REC) and Medicines and Health Regulatory Authority (MHRA)(19/SC/0532) and prospectively registered on the National Institute for Health and Care Research (NIHR) Clinical Research Network (CRN) portfolio (CPMS ID 44473) and the EU Clinical Trials Register (2019‐003468‐27). The trial protocol is attached as Appendix S1 and the CONSORT [6] guidance checklist as Appendix S2. The study design, trial protocol and patient information leaflet were developed with the input from a patient focus group to ensure relevance, acceptability and accessibility for participants. Trial oversight was undertaken by an independent trial steering committee, and the authors vouch for the completeness of the data and the fidelity of the trial.

### 3.2 Participants

We included couples with UI (regular intercourse and failure to conceive for at least 12 months) where the female partner was up to 42 years of age, BMI ≤ 30 kg/m^2^ and with investigations from within the last 12 months demonstrating:

- Bilateral tubal patency confirmed through hysterosalpingography (HSG)/ hystero‐salpingo‐contrast sonography (HyCoSy)/laparoscopy.
- Ovulation is confirmed by either a history of regular menstrual cycles, corpus luteum/dominant follicle seen on ultrasound scan or mid‐luteal serum progesterone over 30 nmol/mL.
- SA with: > 15 mil/ml concentration, motility over 40%, progressive motility > 32% and ≥ 4% normal forms.
- No obvious uterine cavity anomalies/pathology detected on 3D ultrasound
- No erectile dysfunction or other problems preventing penetrative intercourse.

Eligibility was confirmed for all participants prior to randomisation.

### 3.3 Recruitment, Consent and Randomisation

All couples who presented following GP referral for fertility treatment from February 2020 to December 2021 were screened for eligibility and invited to participate in the trial. After we had confirmed they met the inclusion criteria, and following informed consent, we randomised participants in a 1:1 ratio to the control or intervention arms, as detailed below, using sealed opaque envelopes by a researcher blinded to the allocation sequence. No stratification or restriction factors were used for the randomisation.

### 3.4 Intervention

We randomised participants to either the control group (A) (no treatment) or intervention group (B) (progesterone 400 mg twice per day) for participation over three menstrual cycles.

All couples were provided with digital ovulation detection kits (Clearblue, SPD Spark Ltd) used according to manufacturer's instructions depending on cycle length and used once a day in the mornings. We instructed participants to record and report via email/telephone the first day of their menstrual cycle and the date of a positive ovulation test (luteinising hormone (LH) surge). All participants were given advice relating to the fertile period of the menstrual cycle and attempting to achieve pregnancy.

The intervention group additionally commenced vaginal administration of 400 mg bd micronised progesterone pessaries (Cyclogest, LD Collins Ltd) for 14 days starting 24h after a positive LH surge detection.

Participants in the control group tested for pregnancy at home if menstruation was delayed. All intervention‐arm participants were instructed to take a urine home pregnancy test (SPD Spark Ltd) on the 15th day after the positive LH surge. They were instructed to continue the pessaries for a further 38 days if the test was positive. After a negative pregnancy test, they were instructed to stop progesterone and await menstruation.

All pregnancies with a positive pregnancy test were followed up with an early pregnancy ultrasound scan at 6–7 weeks of gestation, and couples were contacted for pregnancy outcomes via email/telephone after delivery.

The TIDieR template for intervention description and replication is attached as Appendix S3.

### 3.5 Data Collection

Patients used email and telephone to secure NHS email addresses and telephone numbers to provide their data, which was stored in a secure anonymised database. Contact was made with participants at least once per cycle to ensure timings were documented and adherence to protocol confirmed.

### 3.6 Outcome Measures

The primary outcome was livebirth, defined as the birth of a live baby and multiple births recorded as a single livebirth event. Secondary effectiveness outcomes included biochemical pregnancy rate, clinical pregnancy rate and mid‐luteal serum progesterone level. Biochemical pregnancy was defined as a positive urinary human chorionic gonadotrophin (hcg) test on the 15th day after the LH surge. Clinical pregnancy was defined as ultrasound confirmation of gestational sac and/or heartbeat (International Committee for Monitoring Assisted Reproductive Technologies ICMART) [7]. The recommended core outcome sets for research in infertility include these outcome measures [8] and the patient involvement group who assisted in the early planning of the trial agreed on the importance of these.

Safety outcomes included miscarriage and ectopic pregnancy rates (further defined as biochemical pregnancy loss rates (prior to clinical pregnancy), early miscarriage rates (clinical pregnancy to 12 weeks’ gestation), late miscarriage rates (12–24 weeks’ gestation) and stillbirth rates (after 24 completed weeks of pregnancy)). We were unable to perform a serum progesterone level for most participants due to restrictions on hospital attendances during the COVID‐19 pandemic.

### 3.7 Statistical Analysis

Prospective sample size calculation was based on the primary outcome of live birth. Seventy couples were required for each group (140 in total) to provide 80% power to detect a 17% absolute difference in live births from 7% to 24% between the control and treatment groups at a two‐sided alpha level of 0.05. The expected live birth rate in the control arm with no treatment was 7% [9]. In the absence of similar trials, the effect size was based on a related study that trialled a different intervention but reported outcomes following luteal phase progesterone in a control group. Considering a generous 30% loss to follow‐up, we aimed to recruit 200 participants in total.

### 3.8 Statistical Analysis

Baseline and demographic data were summarised by means (standard deviation (SD)) or medians (minimum, maximum, interquartile range (IQR)) for continuous variables and as numbers (%) for categorical variables [10, 11]. All outcomes were analysed based on the intention‐to‐treat principle. For each of the outcomes, we presented the treatment effect as a risk ratio (RR) along with a 95% confidence interval and a two‐sided *p* value.

Additionally, we performed univariable logistic regression for the primary outcome for potential confounding variables: anti‐mullerian hormone (AMH), antral follicle count, parity and presence of anovulatory cycles to reconfirm our estimate of the intervention effect.

Data were analysed using Stata analysis [10] and SPSS [11] software. The final statistical analysis plan was accepted as a protocol amendment by the REC and MHRA before data lock and analysis.

## 4 Results

### 4.1 Trial Participants

The trial recruited participants from February 2020 to December 2021 with a pause from March to September 2020 due to the closure of the fertility services due to the COVID‐19 pandemic.

A total of 268 couples were identified during this time as possibly meeting the eligibility criteria.

Fifty‐two were subsequently found to be ineligible after either SA or tubal patency testing, 49 did not complete their infertility investigations, and 24 declined recruitment. One hundred and forty‐three couples were recruited.

**FIGURE 1:**
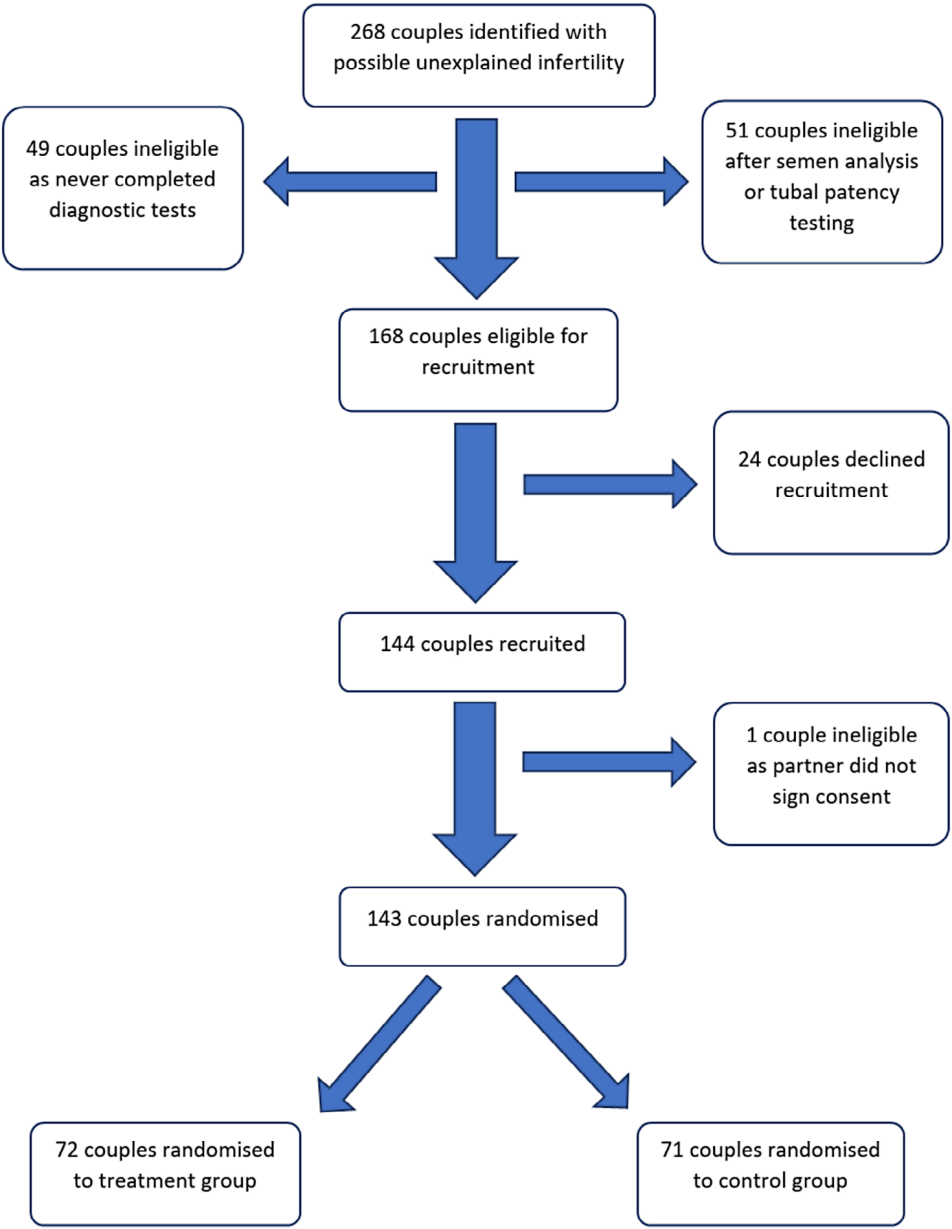
Recruitment process results.

Of 143 couples randomised, 72 were randomised to the intervention group and 71 to the control group. No participant withdrew consent after randomisation. No participant was lost to follow‐up (Figure 1).

Considering the pandemic‐related delays, low dropout rates during the trial and limited resources, a decision was made by the trial team and supported by the Trial Steering Committee for early termination of the trial when the minimum requirement of 140 participants was met.

Data collection started in March 2020 and continued until the final livebirth was recorded in November 2022 when the trial was closed. A Gantt chart for trial progress and graph of expected versus actual recruitment are attached as Appendix S4 and S5.

Figure 1. Recruitment process.

Baseline demographic and clinical characteristics of participants in the two groups were similar. The mean age of participants was 34.1 years (treatment) and 34.4 years (control). The median body mass index (BMI) was 22 and the median time trying to conceive was 2 years in both groups (Table 1).

**TABLE 1:** Baseline variables.

Variable (female partner)	Treatment group (*n* = 72)	Control group (*n* = 71)
Age (years)	34.1(3.92) *n* = 70	34.4 (3.93) *n* = 71
BMI (kg/m2)	22 (18–30) IQR 21–25 *n* = 65	22 (18–30) IQR 20.2–25 *n* = 63
Anti‐mullerian hormone level (pmol/L)	16.7 (3.8–66.5) IQR 9.3–29.7 *n* = 68	15.7 (3.2–103.9) IQR 9.5–26 *n* = 66
Mean antral follicle count (n)	17.5 (3–63) IQR 13–28 *n* = 66	17.5 (5–54) IQR 11–26 *n* = 66
Cycle length (days)	28.4 (2.8) *n* = 57	28.0 (1.7) *n* = 56
No. participants with ≥ 1 previous livebirth	13 (18.8)	4 (6)
No. participants with ≥ 1 previous pregnancy loss	20 (27.8)	18 (25.4)
No. of participants with a history of medical conditions	32 (44.4)	22 (31)
No. of participants taking any medication	10 (13.9)	10 (14.1)

Variable (male partner)		
Age (years)	36.4 (4.9) *n* = 63	35.8 (4.9) *n* = 64
Sperm density (mil/ml)	68 (20–472) IQR 33–122.5 *n* = 59	52 (6–222) IQR 34–107.5 *n* = 56
Sperm progressive motility (%)	48.4 (9.9) *n* = 59	49.6 (10.1) *n* = 57
Sperm normal forms(%)	4 (4–8) IQR 4–5 *n* = 58	4.5 (1.0) IQR 4–5 *n* = 54
Previous livebirth	6 (10.1) *n* = 59	6 (10.7) *n* = 56

Variable (couple)		
Time trying to conceive (years)	2 (1–7) IQR 1.5–2.0 *n* = 65	2 (1–7) IQR 1.5–3 *n* = 64

*Note:* Mean (SD), Median (Range, IQR), *n* (%).

### 4.2 Outcome Measures

Live birth data were available for 143 (100%) participants in the study. Livebirth rates were 11/72 (15.3%) in the intervention arm compared to 5/71 (7.0%) in the control arm (RR 2.17 95% CI 0.79–5.93, *p* = 0.13). The absolute risk increase (ARI) for livebirth was 8.23%. Biochemical pregnancy rate was 15/72 (20.8%) in the intervention arm compared to 10/71 (14.1%) in the control arm (RR 1.48 0.72–3.07 *p* = 0.29 ARI 6.7%). Clinical pregnancy rate was 14/72 (19.4%) in the intervention arm compared to 9/71 (12.7%) in the control arm (RR 1.53 (0.71–3.31) *p* = 0.28 ARI 6.7%).

Pregnancy loss was 4/15 pregnancies (26.7%) in the intervention arm and 5/10 (50%) in the control arm (RR 0.53 (0.19–1.51) *p* = 0.24) (Table 2).

Serum mid‐luteal progesterone levels were not tested on a majority of participants due to pandemic‐related restrictions on hospital attendance.

**TABLE 2:** Outcome measures and safety data.

Outcome	Treatment group (*n* = 72)	Control group (*n* = 71)	Risk ratio (with 95% Confidence intervals)	*p*
Primary outcome				
Livebirth (%)	11 (15.3)	5 (7)	2.17 (0.79–5.93)	0.131
Secondary outcomes				
Biochemical Pregnancy/Positive Pregnancy test (%)	15 (20.8)	10 (14.1)	1.48 (0.72–3.07)	0.293
Clinical pregnancies (%)	14 (19.4)	9 (12.7)	1.53 (0.71–3.31)	0.277
Safety outcomes (all reported as total number and % of total biochemical pregnancies)	*n* = 15	*n* = 10		
Total pregnancy loss rates	4 (26.7)	5 (50)	0.53 (0.19–1.51)	0.238
Biochemical loss (< 7 weeks gestation)	1 (6.7)	1 (10)	−0.67 (0.05–9.47)	0.765
Miscarriage (1st trimester)	2 (13.3)	3 (30)	0.44 (0.09–2.2)	0.321
Total miscarriage rates (miscarriage and biochemical loss)	3 (0.2)	4 (0.4)	0.38 (0.06–2.24)	0.28
Ectopic pregnancy	1 (6.7)	0 (0)	2.06 (0.09–46.12)	−0.648
Termination of pregnancy for medical reasons	0 (0)	1 (10)	−0.23 (0.01–5.12)	0.353

The univariable logistic regression analysis to evaluate the effect of AMH, AFC, non‐ovulatory cycles or parity on the primary outcome did not demonstrate any significant effect on results.

The trial reported 2 (1.4%) adverse events, both of which were considered unrelated to the trial. One was a growth‐restricted baby delivered prematurely due to reduced growth velocity possibly related to severe COVID‐19 infection in the first trimester, and the other was an antenatal diagnosis of Patau syndrome which resulted in a termination of pregnancy.

The trial reported 16 (11.2%) protocol deviations, including allocation nonadherence. Fifteen couples started alternate fertility treatments during the trial period, and one couple used basal body temperature testing as well as ovulation kits and timed starting treatment using this method.

### 4.3 Additional Subfertility Factors

Twenty‐one participants reported 30 cycles without an LH surge detectable using the digital ovulation kits. The mean cycle length for these cycles was 29 days. This could not be confirmed with a serum progesterone measurement due to the restrictions of the COVID‐19 pandemic. Those in the treatment group were advised not to use progesterone in those cycles. These cycles all ended with a menstrual bleed, and participation in the trial resumed in the subsequent cycle.

Despite meeting the initial inclusion criteria, three women were diagnosed with endometriosis and one with ovulatory polycystic ovary syndrome (PCOS) due to further investigations during the trial period.

## 5 Discussion

### 5.1 Main Findings

This trial was unable to demonstrate a significant increase in livebirth rates with exogenous luteal phase progesterone supplementation compared to no treatment in couples with UI. There were no significant differences in biochemical pregnancy, clinical pregnancy or miscarriage between the groups.

### 5.2 Clinical Implications

To our knowledge, PINC is the first RCT to evaluate the effectiveness of exogenous luteal phase progesterone supplementation in otherwise unmedicated natural cycles. Consequently, the estimation of an effect size was based on indirect evidence and resulted in the inability to demonstrate significant differences between arms for the prospectively calculated sample size. The trial, however, demonstrated feasibility for a large‐scale trial as the intervention was acceptable to women, with excellent rates of recruitment and retention, and allowed us to estimate an effect size for the planning of a larger trial. The 8.3% absolute increase in livebirth rates from 7.0% to 15.3% and a similar increase in biochemical and clinical pregnancy rates reflect a clinically important increase requiring further investigation.

If reproducible, a 15.3% cumulative livebirth rate over 3 months is similar to the cumulative success rates reported for stimulated IUI treatment [12]. Stimulated IUI is not only expensive but also invasive, and the use of luteal phase progesterone alone was more acceptable to our patient focus group. No participants dropped out of the trial due to medication side effects.

Despite fulfilling the inclusion criteria, four women were diagnosed during the trial period with endometriosis or polycystic ovarian syndrome. Unless deeper testing for these conditions is standardised, women with ovulatory PCOS or asymptomatic endometriosis with unaffected tubes or other underlying conditions will be included in the UI category. The existence of luteal phase defect in women with UI is unproven, but the possibility of reduced progesterone‐mediated receptivity or a delay in reaching optimal receptivity has been suggested [4]. Progesterone resistance is a phenomenon seen in both PCOS and endometriosis, and it seems plausible that it could also affect women within the UI cohort, especially if some proportion of them may have these undiagnosed conditions.

Micronized vaginal progesterone has a long history of usage in assisted reproductive technology (ART) cycles and more recently in miscarriage prevention and has a proven safety record. The exact mechanism of action of exogenous progesterone to improve receptivity in UI is unproven but progesterone receptor upregulation can be mediated by other steroid hormones including oestradiol and androgens [13].

The underlying mechanism for how exogenous progesterone can improve receptivity in a natural cycle with normal endogenous production remains unclear. We suggest there might be an upregulation of the progesterone receptors and a positive feedback on the corpus luteum [14] via the nonclassical progesterone receptors [15, 16] which leads to further release of endogenous progesterone as well as relaxin and oxytocin. Other suggested mechanisms are that the exogenous progesterone influences epigenetic changes leading to improved receptivity [17] or that exogenous progesterone helps downregulate Cyclin E expression in the endometrium [18]. This needs to be further researched as decades of research on the luteal phase are yet to culminate in a clear answer.

### 5.3 Research Implications

A further larger scale multicentre RCT, powered to detect the reported effect size for live birth rate, is planned. Future research should also focus on the optimal timing to start progesterone, the optimal dose and the duration of treatment.

### 5.4 Strengths and Limitations

This is the first clinical trial to assess the possibility that luteal phase progesterone support in natural cycles could increase the livebirth rates for women with UI. This randomised trial had robust methodology and addresses an important clinical question. The principles of clinical trial integrity as defined by an international, multi‐stakeholder consensus were adhered to [19]. Although the trial was unblinded, we consider this to have a minimal impact due to the objective nature of the outcome measures [20]. It was not possible for the patients or clinicians to be blinded to the outcomes since there was no placebo planned and the same clinical team members recruited and collected data. It may be hypothesised that the suppositories may produce a placebo effect through unknown mechanisms or that participants in the non‐placebo control arm may have altered behaviour patterns and reduced frequency of sexual intercourse. These uncertainties could be addressed in future trials.

Some of the semen analyses were conducted at external laboratories and some results were not available as raw data. We have accepted these results as they were reviewed and confirmed by the clinician within normal reference ranges according to WHO laboratory reference ranges but not transcribed to medical records.

It may be argued that a pragmatic trial design may introduce bias due to differences in arms for the several variables that may impact live birth. However, due to the randomised design of the trial, these variables are randomly distributed between arms and hence the existing differences do not increase bias. Additionally, we performed a univariable regression analysis to assess the impact of the confounding variables on the outcome. This has reported no significant differences.

We acknowledge that this study was, with hindsight, underpowered due to the lack of relevant literature to estimate the effect size, but the reported effect size provides valuable insight for planning future trials. A post hoc power analysis was performed which showed that with 70 participants in each group and 7% livebirths in the control group compared to 15% in the treatment group, α was 0.05 and the power was 33%. The follow‐up trial will have a placebo arm to minimise bias and effects on participant behaviour and will be powered to an estimated treatment effect size based on these results. The trial was run in a single centre with a small research team which minimised performance bias and differences in the care and support offered to participants. For the 143 recruited and randomised couples, none were lost to follow‐up due to the single‐site nature of the trial; all recruited couples who did not conceive remained under the care of the Homerton Fertility Unit. The regular contact with participants also improves our confidence regarding trial adherence ‐ excluding those who underwent IVF or IUI cycles as protocol deviations, no reports were made of the use of other fertility medications or lack of intercourse in either group. Despite being a single‐centre trial, the population served by this Fertility Unit is diverse regarding age, ethnicity and socio‐economic status.

It is important to note that exogenous progesterone may mask the symptoms of early pregnancy problems, including bleeding and an early pregnancy scan should be performed for all women on treatment to prevent delayed diagnosis of miscarriage or ectopic pregnancy.

Patients did not all continue their antenatal care in the same hospital setting; therefore, pregnancy outcomes were self‐reported. The outcomes are objective and, other than the biochemical pregnancy rate, were all confirmed by medical practitioners; therefore, self‐reporting should not have influenced the results.

The starting point of progesterone supplementation was chosen to balance participant convenience and to mimic the timing of endogenous production. Ovulation testing was performed once each morning, and this timing was chosen as it represents a range from 24 to 47 (mean 36) hours after the actual LH surge occurred. This mimics ovulation and also endogenous progesterone production, for which there is growing evidence that this begins before oestradiol levels peak and the LH surge [21]. This timing also optimised the simplicity of the protocol, encouraging compliance.

Mode of administration, dose and duration were based on the licensed usage within ART cycles.

## 6 Conclusions

In conclusion, this trial is the first RCT assessing the value of luteal phase progesterone support during natural cycles for women with UI. This trial was unable to detect a significant difference in live birth rate with luteal phase progesterone treatment in natural cycles compared to controls. However, the results demonstrate feasibility to warrant further research and an effect estimate for planning future sample sizes. Progesterone supplementation has the potential to provide a simple, inexpensive, patient‐friendly, safe and noninvasive treatment option for UI.

## Author Contributions

Claudia Raperport conceived the idea for the trial and performed the background research and literature review. Claudia Raperport developed the protocol and set up and ran the trial under the supervision of Priya Bhide and Roy Homburg. Elpiniki Chronopoulou assisted Claudia Raperport in the recruitment of participants and Sheetal Barhate with the early pregnancy scanning. Elizabeth Timlick and Kristina Sackett helped with trial administration. Aviva Petrie performed all of the statistical analysis. All the authors contributed to the final manuscript. The corresponding author attests that all listed authors meet authorship criteria and that no others meeting the criteria have been omitted. The anonymised raw data for this trial and statistical outputs will be available on request.

## Conflicts of Interest

The authors declare no conflicts of interest.

## Supporting information


Data S1.

Data S2.

Data S3.

Data S4.

Data S5.

## Data Availability

All anonymised individual participant data will be available upon request. Data will include demographic data and all recorded results/outcomes from the trial. The study protocol with approved amendments including the full statistical analysis plan is attached in appendix s1. Data will be available for 10 years after publication. Data will be shared upon request to the corresponding author, with researchers who provide a methodologically sound proposal, for individual participant data meta‐analysis.
