# Supplementary material for: Progesterone Luteal Support in Natural Cycles for Unexplained Infertility: A Randomised Controlled Trial (The PiNC Trial)
Source: BJOG. 2025 Apr 21;132(9):1220–7. doi: 10.1111/1471-0528.18171 (PMC12232507; doi:10.1111/1471-0528.18171)
Supplement: Supplementary file 1 — Data S1. [file BJO-132-1220-s005.docx]

Full Title: A study of the effects of **P**rogesterone for luteal support **i**n **N**atural **C**ycles for unexplained infertility

Short Title/Acronym: PiNC (Progesterone in Natural Cycles)

IRAS Number:

REC Reference Number:

Chief Investigator: Dr Claudia Raperport,

Homerton University Hospital,

And Queen Mary University of London

Glossary of terms and abbreviations:

HSG - Hysterosalpingogram

HyCoSy – Hysterosalpingo - Contrast - Sonography

hCG – Human chorionic gonadotrophin

ART – Assisted Reproductive Technology

LH – Luteinising Hormone

FSH – Follicle Stimulating Hormone

AMH – Anti-Mullerian Hormone

ERα – Estrogen Receptor α

GdA – Glycodelin-A

HMG – Human Menopausal Gonadotrophin

SA – Semen Analysis

Contents:

Page 3 - Project Synopsis

Page 4 -5 – Introduction

Page 5 - Trial Objectives

Page 5-6 – Methodology

Page 7-9 – Study Procedures

Page 9 – Results Analysis

Page 9-10 Data Management and Access

Page 10 – Ethics

Page 10 - Data Handling and Record Keeping

Page 10-11 Safety Reporting

Page 11-12 Trial Committees

Page 12 – Finance and Funding

Page 13 – Dissemination of Research Findings

Page 13 - References

Summary/Synopsis

| Short Title | Progesterone in Natural Cycles |
| --- | --- |
| Methodology | Single centre, parallel arm randomised controlled trial. Following informed consent, participants will be randomised to 3 cycles of treatment or no treatment. Primary outcome is live birth rate.  Each month participants will be asked to record the first day of their menstrual period. On the relevant cycle day according to the test instructions they will start doing daily LH monitoring using a quantified validated test (same brand) until a positive result is seen. 24hrs later they will start 400mg bd progesterone (Cyclogest) pessaries (vaginal or rectal) and continue these for 14 days. After 7 days they will attend for a serum progesterone level blood test. They will then check a urine pregnancy test (validated, same brand) on the 14^th^ day after starting cyclogest (15^th^ day after LH surge). They will discontinue the progesterone if hcg is negative and those who are pregnant will continue progesterone for 38 days. 2 weeks later they will be offered an early pregnancy ultrasound.  We will gain consent to check NHS records to record live births 42 weeks after the positive pregnancy test. We will ask patients to contact us if they have a miscarriage or late pregnancy loss but will not contact them to chase for information as this does not directly impact on our measurable outcomes (clinical pregnancy rates and live birth rates). |
| Research Sites | Homerton University Hospital |
| Objectives/Aims | To assess whether luteal support with progesterone pessaries affects the likelihood of clinical pregnancy and/or live birth in natural cycles for couples with unexplained infertility |
| Number of participants/patients | 200 in total – 100 in the test group and 100 in the control group |
| Main Inclusion Criteria | Female:   - bilateral patent tubes within last 12 months (HSG/HyCoSy/Laparoscopy) - Ovulation as defined by regular cycles or ultrasound appearance of either a corpus luteum or dominant follicle or midluteal progesterone over 30nmol/ml - Regular intercourse and failure to conceive for at least 12 months   Male:   - Semen analysis with: - >15 mil/ml concentration - Motility over 40% - Progressive motility over 32% - ≥ 4% normal forms - No erectile dysfunction or other problem preventing penetrative intercourse |
| Statistical Analysis & Methodology | Using a baseline expected live birth rate of 7% and an expected live birth rate of 24% in the treatment group, with an α0.05 and 80% power, we need to recruit 70 participants to each group. Allowing for some drop-outs, we will aim to recruit 100 per group. |
| Proposed Start Date | November 2019 |
| Proposed End Date | April 2021 |
| Study Duration | 18 months |

**Introduction:**

Background:

Unexplained infertility affects up to 40% of couples referred to fertility services)^1^. Many theories exist regarding the aetiology of subfertility in these couples; however it is well accepted that the underlying cause is likely to be multi-factorial. The challenges faced with unexplained infertility highlight the need to better understand the aetiology and thus improve treatment outcomes and prognosis.

To be diagnosed with true unexplained infertility, a couple must have proven ovulatory menstrual cycles, patent fallopian tubes and an adequate semen analysis (count, motility and normal forms).

With these parameters tested, and assuming regular sexual intercourse, the current school of thought is that the problem arises at the point of implantation rather than fertilisation. There are however no adequate methods of testing successful fertilisation and thus proving this hypothesis.

Progesterone is a steroid hormone produced initially by the corpus luteum after ovulation and then by a developing early pregnancy. It maintains the oestrogenised, thickened endometrium during the luteal phase of the menstrual cycle and stimulates molecular changes and expression of various receptors and molecules that enhance receptivity of the endometrium to implantation of a developing embryo. Progesterone levels are known to peak seven days after ovulation and then in non-conceptual cycles, slowly decline until the onset of menses. In the UK, it is widely accepted that a progesterone level of 30nmol/L in the mid-luteal phase or on day 7 post-ovulation (usually referred to as day 21 assuming a regular 28-day cycle) indicates successful ovulation. In unstimulated cycles, the level of progesterone on day 21 can vary widely as its release is pulsatile and therefore levels can change over a period of a few hours making a single reading difficult to interpret ^2^.

The relationship between progesterone and endometrial receptivity is multi-faceted. The endometrium is designed to undergo many changes through the menstrual cycle. Estrogen receptor alpha (ERα) is downregulated after ovulation by rising serum levels of progesterone. This allows expression of glycodelin-A (GdA) molecules which are involved in implantation processes. The down-regulation of ERα also allows expression of β3 integrin, a cell-to-cell adhesion molecule also important in implantation and endometrial receptivity.

It has been indicated that in the endometrium of women with unexplained infertility that there is a loss of this progesterone-mediated down-regulation process of the estrogen receptors. This then leads to a reduction in GdA ^3^and β3 integrin^4^ expression, both of which are important for optimising endometrial receptivity and the implantation process.

Rationale:

There is a clear need to investigate the role and effect of progesterone in women with unexplained infertility. We hypothesise that a proportion of women suffering with unexplained infertility have either reduced progesterone production or increased progesterone resistance/ decreased progesterone sensitivity. This in turn affects endometrial receptivity which may reduce the likelihood of implantation.

This randomised controlled trial will be conducted in women with unexplained infertility comparing luteal phase progesterone support in natural cycles with no treatment. Primary outcome measures will be live birth rates. Secondary outcome measures will be clinical pregnancy rates and mid-luteal progesterone levels. To our knowledge, no study has been conducted to date investigating the effect of progesterone support in natural cycles.

Existing Research:

No study has investigated progesterone in natural cycles before. However, one study looking into the effect of ovarian stimulation with or without progesterone support had a control arm which received progesterone in natural cycles. This study, where the inclusion criteria demanded a history of infertility of at least 1 year, showed a 24% live birth rate after treatment for up to 3 cycles^5^. A study comparing luteal phase progesterone support across cycles of IUI using clomiphene, letrozole or clomiphene or letrozole plus gonadotrophins, showed an overall clinical pregnancy rate of 24.1% with progesterone and 14.1% without^6^.

Why this research is urgently needed:

Unexplained infertility affects 40% of couple seeking fertility treatment^1^. If one of the underlying factors is a lack of progesterone or increased progesterone resistance, then the addition of exogenous luteal phase progesterone may aid implantation and improve clinical pregnancy rates and live birth rates.

This intervention is simple, low-risk and inexpensive. Compared to the current treatment options for unexplained infertility, this is a non-invasive and inexpensive intervention.

**Trial Objectives:**

Primary objectives:

The primary objective of this study is to determine whether progesterone luteal phase support results in higher live birth rate than no intervention in natural cycles for couple with unexplained infertility.

Secondary Objectives:

Secondary objectives will be to measure any differences between mid-luteal progesterone levels, and clinical pregnancy rates between the treatment and control groups.

**Methodology:**

Study design:

This study is a prospective, pragmatic, two-armed, randomised controlled trial. The hypothesised benefit of the luteal phase progesterone support is to enhance endometrial receptivity and improve conception rates measured as clinical pregnancies and eventually live births.

The study does not aim to validate different formulations of progesterone or different dosing regimens. The design of the trial supports the use of progesterone as it is licensed to be used in ART cycles, at a dose of 400mg twice per day either vaginal or rectal application.

This trial will recruit eligible participants meeting the criteria for a diagnosis of unexplained infertility. All new patients seen in the fertility units will be screened for eligibility and if eligible, will be invited to participate. Following informed consent from both partners, participants will be randomised in a 1:1 ratio to either the treatment or control groups. Women will remain in the trial for three menstrual cycles and will be followed up to the end of the third cycle. If pregnant, the outcome of the pregnancy will be recorded at 6-7 weeks of pregnancy when a scan will be performed to determine clinical pregnancy and subsequently by following up live births within 6 weeks postpartum.

Study Population:

The trial will recruit and randomise 200 participants.

Study setting:

Single centre trial at the Homerton Hospital NHS trust Fertility Unit

Inclusion Criteria:

Female:

- Bilateral patent tubes within last 12 months (HSG/HyCoSy/Laparoscopy)
- Ovulation as defined by regular cycles or ultrasound appearance of either a corpus luteum or dominant follicle or midluteal progesterone over 30nmol/ml
- Regular intercourse and failure to conceive for at least 12 months

Male:

- Semen analysis with:
- >15 mil/ml concentration
- Motility over 40%
- Progressive motility over 32%
- ≥ 4% normal forms
- No erectile dysfunction or other problem preventing penetrative intercourse

Exclusion criteria:

- Age ≥42 (as likely component in infertility),
- Unilateral tubal patency
- BMI ≥30
- Abnormal uterine cavity as seen on 3D ultrasound

Primary outcome:

Live birth rates

Secondary Outcome:

Biochemical pregnancy rates (positive urine HCG test at least 14 days after starting progesterone)

Clinical pregnancy (intrauterine gestational sac confirmed on ultrasound at 6-8 weeks gestation)

Mid-luteal serum progesterone levels

Clinical efficacy outcomes/clinical safety outcomes:

Miscarriage and ectopic pregnancy rates

Stillbirth (pregnancy loss after 24 weeks of gestation)

**Study Procedures:**

Screening:

Potentially eligible participants at the recruiting clinics will be identified at their first appointment. They will already have had a semen analysis and female hormone profile. They may not have had tubal patency testing. In the absence of any risk factors for tubal blockage (known endometriosis, previous pelvic surgery or pelvic infection) they will be given an information leaflet regarding the trial. Prior to tubal patency testing, all hormone results and semen analysis will be checked and eligibility confirmed. If tubal patency has been confirmed prior to this appointment, consent can be taken once the patients have had 24hrs to read the patient information sheet and consider participation.

At the appointment for HSG, HyCoSy or laparoscopy, if tubal patency has been confirmed, patients will be invited to sign a consent form. (HSG results will be formally confirmed with the consultant radiologist after the appointment).

Informed consent:

Written informed consent of both partners will be taken by a trained and delegated member of the study team. This will be done at the time of their tubal patency test or at a follow up appointment.

A copy of the consent form will be given to the participants; one copy will be kept in the woman’s hospital notes and one will be placed in the Investigator Site File. Only members of the research team documented on the delegation log will be able to consent eligible couples for participation in the study. The consenting staff will have thorough knowledge and documented training of research governance issues surrounding consent and will be fully conversant with the study protocol.

The qualified person taking consent must explain to the potential participants that they are free to refuse any involvement within the study or alternatively withdraw their consent at any point during the study and for any reason. If there is any further safety information that may result in significant changes in the risk/benefit analysis, the patient information sheet (PIS) and Informed Consent Form (ICF) will be reviewed and updated accordingly. All participants that are actively enrolled in the study will be informed of the updated information and given a revised copy of the PIS and ICF in order to confirm their wish to continue in the study (if feasible), if it may change their willingness to participate. Participants who speak limited English can only be consented and included in the trial if translation has been provided by an independent translator (i.e. not a family member) or through the Language Line translation service for UK sites. International sites have the option to translate patient-facing documentation into the required language.

Care Pathway:

Couples with unexplained infertility seen at our unit will be offered the option of self-funded IUI treatment or IVF treatment (NHS or self-funded depending on meeting eligibility criteria). Involvement in this trial will be offered to them as an option prior to self-funded treatment or whilst awaiting NHS-funded IVF treatment for which the waiting time is longer than 3 months.

Randomisation:

A computer programme will be used to randomly allocate each trial ID number to a group and patients will be randomly allocated a trial ID number using choice of a paper ticket.

Blinding: It will not be possible to blind the doctors to whether the patients have received treatment or not. The chief investigator will be responsible for organising prescriptions and given the lack of placebo, will know whether the patient has been allocated to the treatment or control group.

Trial Intervention/allocation:

Participants in the treatment arm will receive 400mg Cyclogest pessaries and will be asked to take these twice a day from 24hrs after their LH surge is detected with ovulation kits. 14 days after starting the Cyclogest they will perform a home pregnancy test and inform us of the results. If they are pregnant they will continue Cyclogest for a further 38 days as pregnancy support.

Participants in the control arm will record the time of their LH surge and perform a home pregnancy test 14 days later if their next menstrual period has not started.

All participants will be asked to attend the hospital for a mid-luteal serum progesterone test 7 days after the LH surge is detected in the first cycle only.

Monitoring Adherence

Follow-up:

Participants will be asked to email a trial-specific, secure NHS.net email address with details of their cycles – dates for LH positive results and menstrual period start dates. They will use their trial number rather than identifiable personal data. They will have consented to being contacted by either telephone or email to remind them and ask for their data. They will be reminded a maximum of twice per cycle and if they do not respond it will be assumed that they have withdrawn from the trial.

Start dates of each cycle and each LH surge will be recorded. Participants will attend the phlebotomy department at the Homerton for their mid-luteal progesterone and will have consented to the trial team accessing their records to obtain the result.

Data Collection:

The following data will be collected:

Results from hormone profile and tubal patency tests and semen analysis will have been checked prior to consent – this information would be accessed by the clinical team regardless as part of their clinical care.

Basic demographic data, fertility/ menstrual/gynaecological history, relevant medical history, allergies and medication history, dates of menstrual cycles and LH surges and clinical outcomes.

This data is routinely recorded in a patient’s notes and consent will be obtained to access this. Dates of menstrual cycle and LH surges will be requested from patients during the trial.

Identifiable data will be stored in a password protected recruitment log at site but data entered into the database will be pseudo-anonymised (identified only by a participant’s trial ID).

Participant withdrawal:

Participants will have the option to withdraw at any point during the trial and can do so by contacting the team either by email or telephone.

If at any point during the trial, a participant does not reply to two consecutive requests for results it will be assumed that they have withdrawn and no further attempts at contact will be made.

End of Study Definition:

This will be the date six weeks after the expected date of delivery of the last patient randomised who has an ongoing pregnancy.

**Results Analysis**

Sample size

According to a randomised controlled trial comparing expectant management with stimulated cycle IUI treatment over 3 cycles, 7% of the expectant management group reported live births compared to 24% of the treatment group^7^. The study identified that gave progesterone supplementation in natural cycles also showed a 24% clinical pregnancy rate (live birth rate was not reported).

Using a baseline expected live birth rate of 7% and an expected live birth rate of 24% in the treatment group, with an α0.05 and 80% power, we need to recruit 70 participants to each group. Assuming a drop out rate, we will aim to recruit 100 per group, as was recruited in the Farquhar trial.

Method of Analysis

We have adequate statistical analysis experience within the research team to analyse the data.

Analyses will be intention-to-treat, will include all randomised participants and will analyse according to the treatment group to which they were randomised. For each analysis we will present treatment effect (risk ratio)) along with a 95% confidence interval and a two-side p-value. A P value of <0.5 will be considered statistically significant.

Baseline demographic data will be assumed to be equivalent between the two groups due to the randomisation process.

For each of the outcomes measured (livebirth rate, biochemical pregnancy rates, clinical pregnancy rate, mid-luteal serum progesterone levels and pregnancy loss rates) we will perform a logistic regression, initially doing a univariable regression for each of the potential confounders AMH level, 3 vs no ovulatory cycles according to LH kits, antral follicle count, parity) and combining these together with a variable representing group using lasso or stepwise regression we will perform a multivariable logistic regression analysis.

We will validate the model, looking at calibration and discrimination by providing the c-statistic equivalent to the area under the ROC curve.

**Data management and access**

All data management will be undertaken by the team at Homerton University Hospital. Standard operating procedures will be in place for the collection and handling of data received at the Unit. All study data will be entered into a secure, electronic trial database with restricted access. Data collected on the data collection forms and entered onto the electronic database will only identify the participants by a unique trial number.

Data will be processed on a workstation by authorised staff. The workstations access the network via a secure login and password (password changed regularly). No data will be stored on individual workstations. The Chief Investigator will ensure that this information is kept confidential. All documents will be stored securely and kept in strict confidence in compliance with the Data Protection Act (2018) for sites in the UK.

**Ethics**

The CI will ensure that this study is conducted in full conformity with the current revision of the Declaration of Helsinki (last amended October 2013) on research involving human subjects.

The CI will ensure that the trial is conducted in accordance with all applicable regulatory requirements including but not limited to the Research Governance Framework and the MRC GRP guidelines which are based on ICH Guidelines for GCP, Trust and Research Office policies and procedures and any subsequent amendments.

This trial will only start after approval from a REC, MHRA and confirmation from local R&D department has been obtained. If there is any further safety information which may result in significant changes in the risk/benefit analysis, the Protocol, PIS and ICF will be amended accordingly and submitted to REC for revision and approval.

**Data Handling and Record Keeping**

Confidentiality:

The Investigator has a responsibility to ensure that patient anonymity is protected and maintained. They must also ensure that their identities are protected from any unauthorised parties. Information with regards to study patients from UK sites will be kept confidential and managed in accordance with the Data Protection Act, NHS Caldecott Guardian, principles, The Research Governance Framework for Health and Social Care, and Research Ethics Committee Approval.

Case Report Forms:

For all participants clinical data are routinely recorded in the fertility medical notes or fertility electronic database by clinical staff. Data required for the trial will be recorded on the eCRF from these primary sources. Trained and delegated members of the trial team, as documented on the trial delegation log, will be responsible for the completion of the eCRFs. At randomisation eCRFs will be pseudonymised using a participant code. The code will be generated by the randomisation system and recorded on the Randomisation Form.

Record Retention and Archiving:

During the course of the trial, all records remain the responsibility of the Chief Investigator and must be kept in secure conditions. When the trial is complete, it is a requirement of the Research Governance Framework and Trust Policy that the records are kept for a further 20 years.

**Safety Reporting:**

Adverse events

An AE is any untoward medical occurrence in a trial participant, including occurrences which are not necessarily caused by or related to the trial intervention. An AE can therefore be any unfavourable and unintended sign (including an abnormal laboratory finding), symptom or disease temporarily associated with trial activities.

Due to the high incidence of adverse events routinely expected in this patient population and the low risk of the intervention, only those adverse events identified as serious will be recorded for the trial.

There are no expected adverse events for this intervention.

Serious Adverse events

A serious adverse event (SAE) is defined as an untoward occurrence that: (a) results in death; (b) is life-threatening; (c) requires hospitalisation or prolongation of existing hospitalisation; (d) results in persistent or significant disability or incapacity; (e) consists of a congenital anomaly or birth defect; or (f) is otherwise considered medically significant by the investigator.

The following events are foreseeable in pregnant women undergoing IVF/ICSI and hence will not require reporting as SAEs.

Events relating to the participant:

1. Ovarian hyperstimulation syndrome

2. Pelvic infection/pelvic inflammatory disease

3. Failure of frozen embryos to survive thawing

4. Multiple pregnancy

5. Miscarriage

6. Ectopic pregnancy

7. Hypertensive disorders of pregnancy

8. Gestational diabetes

9. Antepartum haemorrhage

10. Postpartum haemorrhage

Events relating to the baby:

1. Low/very low birth weight

2. Small/large for gestational age

3. Preterm/very preterm delivery

**Trial Committees**

Trial Management

This trial will be sponsored by the Research and Innovation Department at Homerton University Hospital. The trial will be coordinated and managed on a day-to-day basis by the Trial Management Group (TMG) comprising ofDr C Raperport and Miss P Bhide.. TheData monitoring committee (DMC) and Trial Steering Committee (TSC) will provide strategic direction. Appointment of the trial steering committee and data monitoring committee will remain the responsibility of the TMG.

Trial Management Group (TMG)

The Trial Management Group (TMG) will comprise the chief investigator and co-investigator. The TMG will be based at Homerton and will have overall responsibility for the conduct of the trial and will report to the TSC.

Summary Monitoring Plan

The study site will perform trial self-monitoring according to the agreed trust monitoring plan and self-monitoring template. The frequency and intensity will be determined by the Homerton monitoring plan and risk assessment. Trial monitoring will include source data verification checks on informed consent forms and eligibility for randomisation and a sample set of CRFs.

Audit and Inspection

A random sample of cases will be monitored. The documents to be verified will be randomly selected. Any major discrepancies found would trigger a more extensive audit of trial data. In addition, the sponsor may also carry out an internal audit throughout the duration of the trial.

Data Monitoring Committee

A single independent Data Monitoring Committee (DMC) will be established for the trial. It will comprise of an independent chair and at least two further independent members who are experts in the field, such as a clinician, trial methodologist and statistician. The DMC will meet bi-annually. Collaborators and all others associated with the trial may write through the trial office to the DMC, to draw attention to any concern they may have about the possibility of harm arising from the treatment under study, or about any other matters that may be relevant. The DMC will also monitor the progress of the trial and will report to the TSC.

Trial Steering Committee

The trial will be supervised by a single international independent Trial Steering Committee (TSC). The Trial Steering Committee (TSC) will have an independent chair and at least two further independent members. The TSC will meet biannually. The CI, co-applicants, trials coordinator and senior trials manager will be invited to attend the TSC meetings. The specific tasks of the TSC will be:

 To recommend and approve major amendments to the protocol arising during the trial.

 To receive the reports from the TMG and DMC.

 To approve the Statistical Analysis Plan and any changes thereto.

 To resolve problems brought to it by the trial collaborators.

 To approve trial reports and the main paper for publication.

**Finance and Funding:**

Cyclogest is being provided by LD Collins Ltd, the manufacturer of the product. The products will be serialised as per the Falsified Medicines Directive and will then be decommissioned by the Hospital Pharmacy before distribution to patients.

Pregnancy Tests and Ovulation kits are being provided by SPD Development Company Ltd who manufacture these.

**Indemnity:** The Homerton Hospital NHS trust will cover the indemnity for this trial.

**Dissemination of Research Findings:**

1. The CI will have primary responsibility and co-ordinate dissemination of data from this trial. The TMG will plan to effectively disseminate the findings of the research to all participants, and stakeholders.

The clinical trial report and the main manuscript will be reviewed by TSC before publication.

2. Dissemination to clinicians and clinical professional bodies will be through publications and presentations at national and international conferences relevant to the speciality. We aim to publish the findings in the highest impact peer reviewed journals and present them at the annual conferences related to the speciality. We plan to publish the study protocol in an open access journal.

3. A writing committee will be appointed which will follow the authorship criteria used by high impact peer reviewed journals (www.icjme.org). Members of the committee will be named authors on the trial monograph and principal study paper. Other team members with substantial contribution to the trial will be formally acknowledged in publications arising from the trial.

Signed by Chief Investigator: Date:


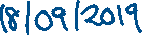

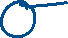

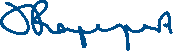


Dr Claudia Raperport, MRCOG

**References:**

1. Smith, S., Pfiefer, SM., Collins, J. (2003) Diagnosis and management of female infertility, *JAMA*, 17(290)
2. Filicori, M., Butler, J.P., Crowley, W.F. Jr, (1984) Neuroendocrine regulation of the corpus luteum in the human: evidence for pulsatile progesterone secretion. *J Clin Invest* 73(6): 1638-1647
3. Dhorostgoal, M., Ghaffari, H., Moramezi, F., Keikhah, N. (2018) Overexpression of Endometrial receptor α in the window of implantation in women with unexplained infertility. *International Journal of Fertility and Sterility* 12(1) 37-42
4. Lessey, B.A. et al, (2006) Estrogen receptor alpha and defects in uterine receptivity in women. Reproductive Biology and Endocrinology (4)
5. Check, JH., Brasile, D., Liss, J., Cohen, R. (2010) A comparison of three types of therapies for three different ovulation disorders in establishing pregnancies and evaluation of laboratory parameters that could influence the outcome. Fertility Sterility 93(5) S11
6. Agha-Hosseini, M., Rahmani, M, Alleyassin, A., Sfadarian, L., Sarvi, F. (2012) The effect of progesterone supplementation on pregnancy rates in controlled ovarian stimulation and intrauterine insemination cycles: a randomised prospective trial (2012) European Journal of Obstetrics and Gynaecology and Reproductive Biology, 165 249-253
7. Farquhar, C.M. et al, (2018) Intrauterine insemination with ovarian stimulation versus expectant management for unexplained infertility (TUI): a pragmatic, open-label, randomised, controlled, two-centre trial. *The Lancet*; [Volume 391, ISSUE 10119](https://www.thelancet.com/journals/lancet/issue/vol391no10119/PIIS0140-6736(18)X0005-1), P441-450,
