# Supplementary material for: Progesterone Luteal Support in Natural Cycles for Unexplained Infertility: A Randomised Controlled Trial (The PiNC Trial)
Source: BJOG. 2025 Apr 21;132(9):1220–7. doi: 10.1111/1471-0528.18171 (PMC12232507; doi:10.1111/1471-0528.18171)
Supplement: Supplementary file 3 — Data S3. [file BJO-132-1220-s004.pdf]

TIDieR

PiNC (Progesterone in Natural Cycles) study

Why: The treatment of unexplained infertility is invasive and expensive. This intervention assesses a simple, safe and inexpensive alternative. Exogenous progesterone may transform the endometrium into a receptive state improving rates of implantation leading to more livebirths.

What (material): A patient information leaflet was given to all participants.

What (procedures): All participants in both groups were provided with Clearblue digital ovulation kits measuring urinary oestradiol and luteinising hormone and an accompanying information leaflet on how to use them.
The treatment group were provided with 400mg micronised progesterone pessaries which were inserted vaginally twice a day starting 24hrs after a luteinising hormone surge was identified on the ovulation test kits.
A clearblue pregnancy test was also supplied for participants in the treatment group for each menstrual cycle during their participation. They tested for urinary hcg (human chorionic gonadotrophin) according to the instructions on the 15th day after starting the treatment.

Who provided: An early pregnancy scan was performed for pregnant participants, performed by a doctor with appropriate experience and at minimum intermediate RCOG scanning module competency.

How (mode of delivery; individual or group): The intervention was self-administered by the participants at home.

Where: The study was run from a tertiary referral NHS Fertility clinic.

When and how much: Participants in the treatment group inserted one 400mg pessary into the vagina twice per day for 14 days starting 24hrs after a positive LH surge was identified. On the 15th day a pregnancy test was performed and treatment continued for a further 38 days if pregnant. If not pregnant treatment was stopped. This continued for a maximum of 3 menstrual cycles.

Tailoring: There was no tailoring of the intervention in this study.

Modification: There was no modification of the intervention in this study.

How well (planned): 15 couples started fertility treatment during the trial period, 14 tried IUI and one IVF. Of these couples seven were in the treatment group and eight in the control group. There were three pregnancies achieved by these couples, one miscarriage and two livebirths, all three were in the treatment group.
Two couples (both in the control group) experienced a breakdown in their relationships and stopped trying to conceive after randomisation.
One couple in the treatment group stopped the progesterone as per protocol due to a negative pregnancy test. After a delay in menstruation they repeated the test which was then positive, did not restart the progesterone and experienced a miscarriage.
The two miscarriages described above were the only miscarriages in the treatment group of clinical pregnancies confirmed on ultrasound (there was one biochemical loss and one ectopic pregnancy).
One couple measured basal body temperature and used this as a guide to starting progesterone and therefore started treatment later in the cycle than the protocol plan.
21 participants experienced one or more cycles without an LH surge detectable using the digital ovulation kits. Of these, 12 were in the treatment group and nine in the control group. Those in the treatment group were advised not to use progesterone in those cycles. Two participants,

one in each group, achieved pregnancies in ovulatory cycles after cycles with no detected LH surge, subsequent cycles, both of which ended in a livebirth

How well (actual): Of the participants who did not revert to alternate fertility treatments, adherence to protocol was 100% and no missed doses or early cessation of treatment was reported.
