# Supplementary material for: Progesterone Luteal Support in Natural Cycles for Unexplained Infertility: A Randomised Controlled Trial (The PiNC Trial)
Source: BJOG. 2025 Apr 21;132(9):1220–7. doi: 10.1111/1471-0528.18171 (PMC12232507; doi:10.1111/1471-0528.18171)
Supplement: Supplementary file 5 — Data S5. [file BJO-132-1220-s002.docx]

The PiNC trial recruitment progress chart – expected versus actual timeline.
